# Physical and mental health predicts better adherence to exercise intervention in older women: A post-hoc analysis

**DOI:** 10.1016/j.heliyon.2024.e32128

**Published:** 2024-05-29

**Authors:** J. Laakso, J. Kopra, H. Koivumaa-Honkanen, J. Sirola, R. Honkanen, H. Kröger, T. Rikkonen

**Affiliations:** a Kuopio Musculoskeletal Research Unit (KMRU), Surgery, Institute of Clinical Medicine, University of Eastern Finland (UEF), Kuopio, Finland; b Institute of Clinical Medicine, Psychiatry, University of Eastern Finland (UEF), Kuopio, Finland; c Mental Health and Wellbeing Center, Department of Psychiatry, Kuopio University Hospital, Kuopio, Finland; d Department of Orthopedics, Traumatology and Hand Surgery, Kuopio University Hospital, Kuopio, Finland; e School of Computing, University of Eastern Finland, Kuopio, Finland

**Keywords:** Adherence, Exercise, Falling, Clinical trial, Women, Aged

## Abstract

**Background:**

Adherence to exercise is crucial for promoting health and maintaining functioning.

**Aims:**

To investigate predictors of adherence to exercise in the initially free supervised fall prevention RCT and its low-cost, self-sustained continuation among elderly women.

**Methods:**

In the 2-year Kuopio Fall Prevention Study RCT, 457 women (aged 71–84) were offered a free initial 6-month supervised weekly training program (gym, Tai Chi) in the municipal facilities. Women's adherence during this period was categorized into high (≥80 %) and low (<80 %). In the next six months, their free access to the premises continued without supervision. For the second year, low-cost access was offered with unsupervised independent training in these facilities. The second-year adherence was based on purchasing(yes/no) a gym card to continue exercising. Information on baseline health, functioning, and lifestyle was obtained by mailed questionnaires and physical tests.

**Results:**

For the first six months, over 60 % of the women had high adherence. Only 26 % continued into the second year. For both follow-up years, active training history was related to better adherence. Initial predictors were related to mental health i.e. having less often fear of falls limiting one's mobility, ability to cope with external, not internal hostility, and being in a loving relationship. In the second year, predictors were related to younger age, having less frequent fear of falls, better functional capacity i.e. better strengths (grip and leg extension) and faster Timed “Up and Go” -test.

**Conclusion:**

Better mental and physical health, better functional capacity and active training background were associated with higher adherence to exercise intervention in older women.

What's new?

Key findings.

- Factors concerning mental health had more often associations than physical factors with better adherence to supervised exercise
- Better functional capacity and more active training background were associated with long-term self-sustained exercising in older women

What this adds to what is known?

- It is possible that mental health aspects might affect more than physical concerning adherence to supervised exercise What is the implication, what should change now?
- Physical and mental health aspects should be considered to increase and optimize adherence to exercise in older women

## 1 Introduction

Long-term habitual physical activity is a significant factor in maintaining health, preventing non-communicable diseases and premature death [1]. Physical exercise has also been shown to be beneficial in managing chronic diseases [2]. Thus, improving physical performance and muscle strength seems to be especially important among the elderly [3]. Community-based group exercise programs have been shown to increase physical activity levels in older age [4]. Physical exercise combined with strength and balance training has been shown to prevent falls among community-dwelling elderly [5,6]. Compared with a health education intervention only, structured, moderate-intensity physical activity programs appear to be more beneficial in reducing major mobility disability among older adults at risk for disability [7], even with comparable costs [8].

Physical exercise is beneficial to mental health. In a meta-analysis of prospective cohort studies (49 studies; N = 266,939), physical activity was found to confer protection against the emergence of depression regardless of age and geographical region [9]. Further, according to a systematic review of 39 RCTs, physical activity was protective for cognitive function in individuals older than 50 years when interventions consisted of aerobic-, resistance-, multicomponent training, or tai chi [10]. In a systematic review article by Shaw et al., 2022 [11], good self-rated mental health predicted better adherence whereas depression was negatively associated with adherence to prescribed exercise with participants aged over 65 years.

Despite the benefits of physical activity, committing older adults to various workout routines is challenging. Better adherence has been shown to improve the long-term effectiveness of physical function in exercise therapies [12]. Multiple factors influence adherence and level of independent exercise. While there are studies regarding adherence to exercise programs and physical activity, no summary can be drawn. In several studies, certain factors predicting better adherence seem to repeat. These are high self-efficacy, better physical condition, and psychological factors such as a positive attitude and previous experiences towards exercise [13].

Adherence can be defined as a degree of attendance rate where the number of sessions attended is divided by the number of sessions offered [14]. Other common measures for adherence are the number of participants completing exercise programs or the number of completed exercise sessions per week [14,15] showing that participants with attendance over 80 % had a reduced risk of falling compared to those with a low level of adherence to fall prevention exercises.

The present study aimed to identify predictive factors for a) the high adherence during the initial free-of-charge supervised 6-month exercise period, and b) during the continuation of self-sustained exercising in the second year without supervision among elderly women randomized in the exercise intervention group. In both periods, women's baseline characteristics, mental and physical health (questionnaire data), and functional capacity (objective tests) were analyzed by adherence status.

## 2 Materials and methods

### 2.1 Participants and recruitment

**Fig. 1:**
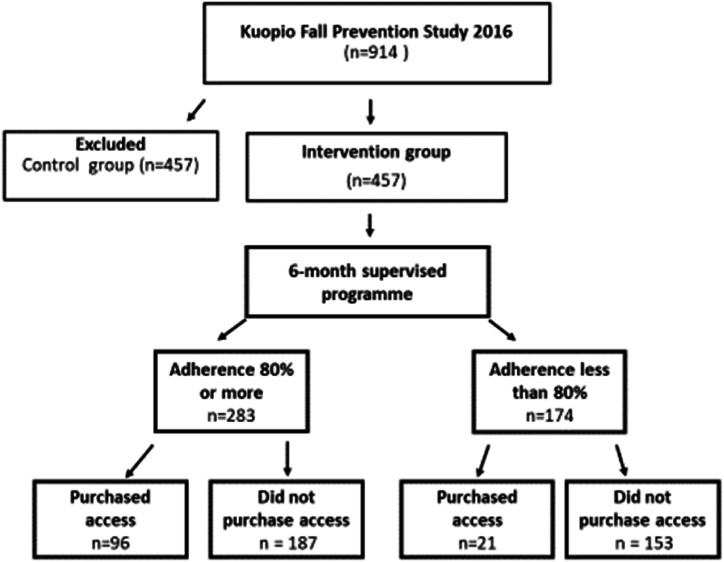
Recruitment and adherence of the study population.

In 2016, a two-year RCT Kuopio Fall Prevention Study (KFPS) [5] was launched in Kuopio City, Finland. It investigates the efficacy of a large, population-based exercise intervention in fall prevention, physical and mental health, and physical functioning of community-living older women (N = 914) born between 1932 and 1945. In the present study, we investigate predictors of adherence in the KFPS intervention group (n = 457) during the two-year follow-up period (Fig. 1).

**Fig. 2:**
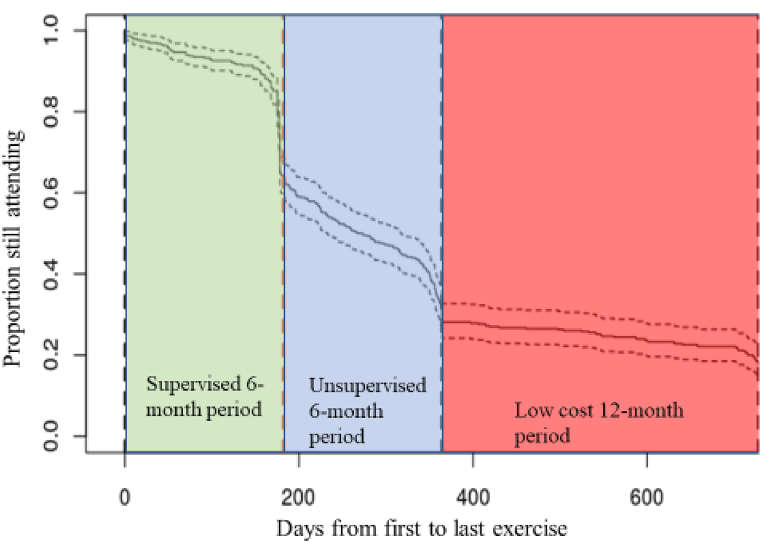
The proportion of women attending to exercise in 24 months (730 days).

The KFPS study subjects were recruited first from the Kuopio Osteoporosis Prevention Study (OSTPRE), which is an ongoing population-based prospective female cohort with data collected at 5-year intervals since 1989. (Fig. 2). The scope of OSTPRE has broadened from bone loss, falls, and fractures to a wider range of health, lifestyle, and psychosocial issues among postmenopausal women. Its target population included all 1932-1941-born women living in Kuopio Province, Finland [16].

In KFPS, the inclusion criteria were women born in 1932–1945, living near Kuopio city center, who had the possibility and ability (adequate health) to attend training sessions twice a week. To reach an adequate sample size in KFPS, an additional recruitment round was organized by mailed invitation letter for these women (Fig. 2). Letter included information for participating and publishing anonymized data. As a result, altogether 582 women in KFPS have previously attended the OSTPRE study.

At the start, KFPS study subjects (N = 914) were randomized into the intervention group (n = 457) and control group (n = 457) (Fig. 2). All of them received health education and fall prevention instructions in the form of lectures and printed material. The first year of the KFPS randomized controlled trial (RCT) on exercise intervention consisted of a free-of-charge, six-month supervised training program including the gym and tai chi exercise followed by six-month unsupervised independent use of exercise facilities. The participants in the intervention group were encouraged to continue their adopted exercise routine from the supervised program, but compulsory training was not required. Independent exercising could be done alone or in a group. Participants were also free to pursue their normal physical activities. Activities outside the intervention were not followed nor included for analysis.

In the second year, an unsupervised low-cost (65€/year) exercise period was offered for the following 12 months in the same exercise facilities. Training took place in the municipal sports facilities of Kuopio city center. The complete KFPS study protocol has been published in detail previously [17].

### 2.2 Study measurements

Data on both physical and mental health and functioning, lifestyle, medication, and sociodemographic factors were collected using a health questionnaire at baseline. Functional tests provided additional data on objective physical functioning.

2.2.1 Sociodemographic and health-related characteristics at baseline included age, weight, height, BMI, marital status, cohabitation, number of prescribed medications, number of diagnosed diseases in the past two years, and perceived overall health (Table 3).

2.2.2 Baseline indicators of physical health and functioning included 1) fall history during the last year (yes/no); 2) ability to ride a bicycle, walk 500 m, run 100 m, carry a 5 kg weight, and climb stairs; 3) duration and frequency of strength, endurance, and utility exercises; and 4) perceived overall fitness (Table 2).

**Table 1:** The mean number of visits (SD) during 1st and 2nd years, according to adherence criteria by intervention adherence (Left) and purchased the access for the 2nd year (Right).

The two-year follow-up	Type and time of exercise period	Adherence criteria				
		Total mean (sd)	low adherence (n = 174) mean(sd)	high adherence (n = 283) mean(sd)	Did not purchase access (n = 340) mean(sd)	Purchased access(n = 117) mean(sd)
**The 1st year**	**Supervised sessions, 6-month** n = 457	18.3 (6.4)	12.2(6.5)	22.0(1.9)	17.5(6.9)	20.7(4.0)
	**Additional visits, 1–6 months** n = 457	16.6(11.2)	12.4(10.8)	19.1(10.7)	14.3(9.2)	23.1(13.8)
	**Independent visits 7–12 months** n = 457	9.6(14.1)	6.4(13.5)	11.6(14.1)	5.3(10.2)	22.1(16.3)
	**All independent visits, 1–12 months** n = 457	26.2(22.6)	18.8(22.0)	30.7(21.9)	19.6(16.7)	45.2(26.5)
**The second year**	**Low cost 13–24 months** n = 457	–	–	–	0	36.0(31.9)

**Table 2:** Adherence according to baseline characteristics of physical health and functioning in the first six months and in the second year.

Physical health and functioning (%)	Adherence criteria						
	Total (N = 457)	According to adherence in the first six months			According to the second-year		
		low adherence n %)	high adherence n (%)	p-value(x^2^)	Did not purchase access n (%)	Purchased access n (%)	p-value(x^2^)
**Falls during the last 12 months?** yes no	171 (38 %) 277 (62 %)	51 (30 %) 118 (70 %)	120 (43 %) 159 (57 %)	**0.007**	123(53 %) 210 (47 %)	48 (42 %) 67 (58 %)	0.361
**Ability to ride a bicycle?** yes hardly no	299 (68 %) 48 (11 %) 92 (21 %)	99 (59 %) 19 (30 %) 51 (11 %)	200 (74 %) 31 (12 %) 39 (14 %)	**0.001**	214 (66 %) 39 (12 %) 72 (22 %)	85 (75 %) 11 (9 %) 18 (16 %)	0.221
**Ability to walk 500m?** yes hardly no	438 (96 %) 14 (3 %) 4 (1 %)	166 (97 %) 4 (2 %) 3 (1 %)	268 (95 %) 12 (4 %) 3 (1 %)	0.466	322 (95 %) 13 (4 %) 4 (1 %)	112 (97 %) 3 (2 %) 2 (1 %)	0.744
**Can you run 100m**? yes hardly no	191 (44 %) 117(27 %) 130 (29 %)	69 (41 %) 40 (24 %) 58 (35 %)	122 (45 %) 77 (28 %) 72 (27 %)	0.181	140 (43 %) 85 (26 %) 100 (31 %)	51 (45 %) 32 (28 %) 30 (27 %)	0.694
**Can you carry 5 kg at least 100m?** yes hardly no	411 (91 %) 31 (7 %) 10 (2 %)	156 (90 %) 14 (8 %) 3 (2 %)	255 (91 %) 17 (6 %) 7 (3 %)	0.628	306 (91 %) 24 (7 %) 7 (2 %)	105 (91 %) 7 (2 %) 3 (1 %)	0.884
**Can you climb stairs?** yes hardly no	429 (95 %) 24 (5 %) 3 (1 %)	158 (91 %) 13 (8 %) 2 (1 %)	271 (96 %) 11 (3 %) 1 (1 %)	0.138	316 (93 %) 21 (6 %) 2 (1 %)	113 (98 %) 3 (1 %) 1 (1 %)	0.305
**Strength training** never 1-3 times/month 1/week 2 or more times/week	196 (43 %) 146 (32 %) 73 (16 %) 40 (9 %)	81 (47 %) 55 (32 %) 25 (15 %) 11 (6 %)	116 (41 %) 90 (32 %) 49 (17 %) 28 (10 %)	0.398	155 (46 %) 105 (32 %) 45 (13 %) 33 (9 %)	42 (35 %) 40 (34 %) 29 (25 %) 6 (6 %)	**0.009**
**Mean duration of strength training,** minutes(sd)	51.8(32.1)	51.1(36.7)	52.3(28.7)	0.804^a^	49.2(32.7)	60.2(28.6)	**0.037**^a^
**Endurance training** never 1-3 times/month 1/week 2 or more times/week	23 (5 %) 18 (4 %) 36 (8 %) 375 (83 %)	9 (5 %) 8 (5 %) 19 (11 %) 136 (79 %)	15 (5 %) 8 (3 %) 18 (6 %) 239 (86 %)	0.235	17 (5 %) 13 (4 %) 29 (9 %) 278 (82 %)	7 (6 %) 3 (3 %) 8 (7 %) 97 (84 %)	0.832
**Mean duration of endurance training,** minutes(sd)	79.7(29.6)	77.6(31.2)	81.0(28.6)	0.302^a^	77.1(28.8)	89.0(30.9)	**0.002**^a^
**Utility exercising** never 1-3 times/month 1/week 2 or more times/week	50 (11 %) 45 (10 %) 36 (8 %) 322 (71 %)	23 (13 %) 17 (10 %) 13 (8 %) 119(69 %)	28 (10 %) 30 (11 %) 22 (8 %) 201 (71 %)	0.739	40 (9 %) 33 (7 %) 25 (6 %) 239 (53 %)	11 (2 %) 14 (3 %) 10 (2 %) 81 (18 %)	0.791
**Mean duration of a utility exercise** minutes(sd)	92.4(82.6)	81.0(67.8)	99.5(90.0)	**0.02**^a^	87.4(75.4)	108.8(101.3)	**0.018**^a^
**Mean duration of a vigorous exercise** minutes (sd)	52.5(34.6)	48.2(31.7)	55.1(36.1)	**0.04**^a^	52.0(35.2)	53.8(32.9)	0.647^a^
**Perceived fitness** poor or very poor moderate good or very good	41 (9 %) 201 (44 %) 215 (47 %)	20 (11 %) 81 (47 %) 73 (42 %)	23 (8 %) 121 (43 %) 139 (49 %)	0.242	33 (9 %) 152 (45 %) 155 (46 %)	10 (8 %) 50 (43 %) 57 (49 %)	0.825

a Independent samples *t*-test (x^2^) chi's square test.

**Table 3:** Adherence according to self-reported baseline sociodemographic and health-related characteristics in the first six months and the second year.

Baseline characteristics (SD)	Adherence criteria						
	Total (N = 457)	According to adherence in the first six months			According to the second-year		
		low adherence n (%)	high adherence n (%)	p-value(x^2^)	Did not purchase access n (%)	Purchased access n (%)	p-value(x^2^)
**Mean Age, yr**	76.4(3.3)	76.7(3.5)	76.3(3.2)	0.381^a^	76.7(3.4)	75.4(3.0)	**0.001**^a^
**Mean Weight, kg (sd)**	69.4 (2.8)	70.3(12.2)	68.9(13.2)	0.279^a^	69.4(13.4)	69.5(11.1)	0.199^a^
**Mean Height, cm**	158,4(7.4)	158.5(5.3)	158.3(8.4)	0.789^a^	158.1(8.1)	159.1(4.8)	0.935^a^
**Mean BMI, kg/m**^**2**^	27.5(4.6)	28.0(4.7)	27.4(5.2)	0.647^a^	27.7(5.1)	27.4(4.2)	0.704^a^
**Marital status, n(%)** single married/cohabiting divorced widow	30 (7 %) 216 (47 %) 75 (17 %) 133 29 %)	7 (4 %) 81 (47 %) 31 (18 %) 54 (31 %)	23 (8 %) 135 (48 %) 44 (16 %) 79 (28 %)	0.461	23 (6 %) 157 (47 %) 54 (16 %) 103 (31 %)	7 (6 %) 59 (50 %) 21 (18 %) 30 (26 %)	0.735
**Cohabitation, n (%)** alone with spouse with children/relative	238 (52 %) 205 (45 %) 13 (3 %)	91 (52 %) 78 (45 %) 5 (3 %)	147 (52 %) 127 (45 %) 8 (3 %)	0.999	180 (53 %) 150 (44 %) 9 (3 %)	58 (50 %) 55 (47 %) 4 (3 %)	0.766
**Prescribed Medication, n (%)** none 1 2 3 4 or more	113 (25 %) 118 (26 %) 97 (21 %) 83 (18 %) 46 (10 %)	45 (26 %) 39 (22 %) 36 (21 %) 30 (17 %) 24 (14 %)	68 (24 %) 79 (28 %) 61 (22 %) 53 (19 %) 22 (7 %)	0.244	85 (25 %) 90 (26 %) 68 (20 %) 60 (18 %) 37 (11 %)	28 (24 %) 28 (24 %) 29 (25 %) 23 (20 %) 9 (7 %)	0.686
**Diagnosed Diseases in the past 2 years, n (%)** none 1 2 or more	349 (76 %) 81 (18 %) 27 (6 %)	126 (72 %) 40 (23 %) 8 (5 %)	223 (79 %) 41 (14 %) 19 (7 %)	0.055	254 (75 %) 65 (19 %) 21 (6 %)	95 (81 %) 16 (14 %) 6 (5 %)	0.351
**Perceived health, n(%)** Very good or good moderate Poor or very poor	227 (49 %) 209 (46 %) 19 (4 %)	80(46 %) 84 (48 %) 10 (6 %)	147 (52 %) 125 (44 %) 9 (3 %)	0.238	159 (47 %) 165 (49 %) 15 (4 %)	68 (59 %) 44 (38 %) 4 (3 %)	0.053

a Independent samples *t*-test (x^2^) chi's square test.

2.2.3 Indicators of mental health and functioning were measured with separate questions and psychometric scales (Table 3). Fear of falling, hostility, and love had each two questions. Both fear of falling and its limitations on day-to-day mobility, as well as both external (having trouble controlling one's temper) and internal hostility (being easily irritated) were assessed. The two love questions included whether a person has someone who loves her/him and whether a person has someone who she/he loves (Table 3). The psychometric scales included: 1) the Life satisfaction scale (LS) [18]; 2) the revised Life orientation test (LOT-R) [19] measuring optimism; 3) Brief resilient coping scale (BRCS) [20]; 4) Perceived stress scale (PSS) [21] and 5) shortened version of Geriatric depression scale (GDS-6) [22] (Table 4).

**Table 4:** Adherence according to self-reported mental health and functioning in the first six months and in the second year.

Mental health and functioning	Adherence criteria						
	Total n (%)	According to adherence in the first six months			According to the second-year		
		low adherence n (%)	high adherence n (%)	p-value(x^2^)	Did not purchase access n (%)	Purchased access n (%)	p-value(x^2^)
**Having fear of falling** n = 457 sometimes/often no	245(54 %) 212(46 %)	101(58 %) 73 (42 %)	144 (51 %) 139 (49 %)	0.136	193(57 %) 147 (43 %)	52 (44 %) 65 (56 %)	**0.021**
**Fear of falling limits day-to-day mobility**, n = 455 no yes	375(82 %) 80 (18 %)	134 (77 %) 39 (23 %)	241 (86 %) 41 (14 %)	**0.029**	279(82 %) 60 (18 %)	96(83 %) 20 (17 %)	0.911
***Being easily irritated, n = 444*** no yes	89(18 %) 355(82 %)	38 (23 %) 125 (77 %)	41 (15 %) 230 (85 %)	**0.032**	62 (19 %) 260 (81 %)	17 (15 %) 95 (85 %)	0.336
***Losing temper easily n = 437*** no yes	414(95 %) 23(5 %)	151 (79 %) 14 (21 %)	263 (97 %) 9 (3 %)	**0.019**	19 (6 %) 305 (94 %)	4 (3 %) 113 (97 %)	0.341
**Having someone who loves, n=421** no yes	38 (9 %) 383 (91 %)	22 (14 %) 140 (86 %)	16 (6 %) 243 (94 %)	**0.010**	30 (10 %) 281 (90 %)	8 (7 %) 102 (93 %)	0.455
**Loving someone, n=430** no yes	27 (6 %) 403 (94 %)	17 (10 %) 150 (90 %)	10 (4 %) 253 (96 %)	**0.008**	21 (7 %) 299 (93 %)	6 (5 %) 104 (95 %)	0.679
**Life satisfaction, n=449** satisfied intermediate dissatisfied	92(21 %) 301(67 %) 56(12 %)	34(20 %) 114(68 %) 20(12 %)	58 (21 %) 187(66 %) 36(13 %)	0.949	63(18 %) 236(69 %) 43(13 %)	29(27 %) 65(61 %) 13(12 %)	0.147
**Revised life orientation test, n=411** high optimism moderate optimism low optimism	121 (29 %) 209 (51 %) 81 (20 %)	38(25 %) 82(54 %) 31(21 %)	83(32 %) 127(49 %) 50(19 %)	0.346	86(28 %) 160(51 %) 66(21 %)	35(35 %) 49(50 %) 15 (15 %)	0.227
**Brief resilient coping scale, n=439** high resilient copers moderate resilient copers low resilient copers	96 (22 %) 182 (41 %) 161 (37 %)	31(19 %) 70(43 %) 61(38 %)	65(24 %) 112(40 %) 100(36 %)	0.567	71(22 %) 140(42 %) 121(36 %)	25(24 %) 42(39 %) 40(37 %)	0.848
**Perceived stress scale, n = 429** low stress moderate stress high stress	273(63 %) 153(36 %) 3(1 %)	102(64 %) 58(36 %) 0	171(64 %) 95(35 %) 3(1 %)	0.405	204(62 %) 121(37 %) 2(1 %)	69 (68 %) 32 (31 %) 1(1 %)	0.554
**Geriatric depression scale, n = 435** no depression possible depression	382 (88 %) 53 (12 %)	141 (87 %) 22 (13 %)	241(89 %) 31 (11 %)	0.546	291(88 %) 40(12 %)	91(87 %) 13(13 %)	0.865

(x^2^) chi's square test.

2.2.4 Functional tests included: 1) a single leg stance test with eyes open; 2) squatting down touching the floor with fingertips and getting up (able/unable); 3) isometric leg extension strength (in newtons) on both legs [23]; 4) dominant hand grip strength (in kilograms) with a handheld dynamometer [24]); 5) Timed Up And Go (TUG) -test (in seconds) and 6) postural body sway analysis [23] in four different settings i.e. 6a) normal stance, foot in V-position with eyes open; 6b) normal stance, foot in V-position with eyes closed; 6c) semi tandem stance with eyes open and 6d) semi tandem stance with eyes closed (in square millimeters of sway) (Table 5). Postural sway was analyzed using a 90 % confidence interval for the swaying pattern. Isometric strength tests involved three attempts for both legs where the highest result was selected for analysis. At baseline, all participants underwent a series of functional tests.

**Table 5:** Adherence according to objective functional test in the first six months and the second year.

Objective functional tests	Total (N = 457)	Adherence criteria					
		According to adherence in the first six months			According to the second-year		
		low adherence (n = 174)	high adherence (n = 283)	p-value (a)	Did not purchase access (n = 340)	Purchased access (n = 117)	p-value (a)
**Single leg stance test, eyes open** sec (sd)	15.3 (11.4)	15.13 (11.7)	15.3 (11.3)	0.863	14.8 (11.4)	16.6 (11.5)	0.338
**Squat test: touching the floor and getting** up, n (%) able unable	352 (77 %) 103 (23 %)	127 (73 %) 46 (27 %)	225 (80 %) 57 (20 %)	0.115(x^2^)	260 (77 %) 78 (23 %)	92 (79 %) 25 (21 %)	0.703(x^2^)
**Left leg extension strength**, Newton, (sd)	300.8(76.2)	299.7(80.1)	301.4(73.8)	0.816	296.02 (75.6)	314.5 (76.5)	**0.024**
**Right leg extension strength**, Newton, (sd)	302.8 (77.4)	297.9 (80.74)	305.8 (75.4)	0.3	299.9(75.6)	311.2 (82.4)	0.181
**Grip strength**, kg (sd)	26.6 (5.5)	26.5 (5.8)	26.7 (5.3)	0.726	26.3 (5.4)	27.5 (5.7)	**0.039**
**Timed up and go**, sec (sd)	9.8 (2.0)	10 (2.3)	9.7 (1.8)	0.106	9.9 (2)	9.4 (1.8)	**0.030**
**Body sway, eyes open** (mm^2^) (sd)	172.9(100.0)	161.4(89.9)	179.3(105.5)	0.063	173.7(99.3)	170,6(102.3)	0.774
**Body sway, eyes closed** mm^2^ (sd)	261.1(168.1)	267.1(183.6)	257.4(158.1(	0.552	266.6(170.8)	245.2(158.9)	0.235
**Body sway, eyes open semitandem** mm^2^ (sd)	252.9(144.9)	248.1(141.4)	255.8(147.2)	0.587	258.5(154.1)	236,7(113.8)	0.161
**Body sway, eyes closed semitandem** mm^2^ (sd)	542.4(327.3)	533.1(313.8)	549.6(335.7)	0.612	549.9(325.7)	524.6(322.9)	0.485

a Independent samples *t*-test (x^2^) chi's square test.

2.2.5 The association between exercise adherence, health, and functioning was analyzed by using the above-described baseline characteristics. The adherence to organized sessions [25] during the free six-month supervised program was categorized into two i.e. low (<80 %) and high (≥80 %) adherence. Adherence percent was calculated by dividing the attended sessions by the number of maximum sessions organized. This information was obtained from electronic access data created by the visits using the personal access card [26] to the exercise premises. The exercise intervention was conducted in 27 groups, each consisting of 15–18 women. The maximum number of sessions during free six-month supervised training varied between 23 and 26 sessions due to the random closure of the training facility during seasonal holidays, maintenance, or other overlapping events. Each group's adherence rate was adjusted to match the maximum number of sessions available. Second-year adherence (unsupervised independent training) was analyzed in two categories i.e. those who purchased low-cost gym cards for the consecutive second-year period vs. those who did not. We also compared independent exercise between the groups during the initial six-month period, the second half of the first year, the total first year, and the second year.

### 2.3 Statistical analyses

Characteristics predicting (high or low) adherence among the intervention group were investigated using Pearson's chi-square test for dichotomous variables and independent samples *t*-test for continuous variables. The significance level was set at p ≤ 0.05 and the confidence interval to 95 %. Indistinct answers (e.g. multiple answers or several options chosen) in the questionnaire were excluded from the data. To meet assumptions of the chi-squared test, class variables in the baseline questionnaire which included less than ten responses were combined with the nearest similar one (*e.g.* number of medications, diagnosed diseases, exercising frequency). A total of 457 community-dwelling elderly women randomized into the intervention group were included in the analysis. Data were analyzed using IBM SPSS Statistics 27 and R [27].

## 3 Results

### 3.1 Adherence

During the six-month supervised training (months 1–6), the adherence rate (i.e. the proportion of participants attending ≥80 % or more of offered sessions) was 61.9 % (n = 283). The mean number of attended supervised sessions was 18.3 (Table 1) which equals 70.4 % of total sessions held (18,3 visits/26 visits). Only 14 (3 %) participants were non-compliant with no visits during the supervised first 6-month period. The mean number of additional independent uses of the facilities outside the supervised sessions during this period was 16.6 (Table 1) (2.8 visits per month). Altogether 36 women (7.9 %) had no additional visits in this period. Those categorized into high adherence had on average 7 additional visits compared to those with low adherence (19.1 vs 12.4) (Table 1).

During the second half of the first year (months 7–12) free unsupervised period, exercise frequency dropped significantly to the mean number of 9.6 visits (i.e. 1.6 per month) (Table 1). The mean number of visits was 11.6 in the high adherence group and 6.4 in the low adherence group. In this period, a total of 185 participants of 457 (40.5 %) had no independent visits (Table 1).

For the first 12-month period (months 1–12), the total number of independent visits on-site was recorded. This period included additional visits in the first six-month period and independent free use in months 7–12. The mean number of independent visits was around 26 per the whole year for the whole cohort. Visits among participants with high or low adherence criteria for the same period were around 31 and 19, respectively (Table 1).

During the second year (months 13–24), the number of women who purchased low-cost gym cards for unsupervised training was 117 (25.6 %) out of 457. Within this group, the mean number of visits was 36.0 (SD 31.9, range 1–171) (Table 1). Out of these women, 96 (82 %) were those who had high adherence during the initial six-month supervised period.

Those who purchased access for the second year had more independent visits in every investigated period. Visits during the supervised six-month period were (additional) 23.1 vs 14.3, in the second half of the first year 22.1 vs 5.3. and for the whole first 12-month period 45.2 vs 19.6.

### 3.2 Adherence related to physical health, functioning, sociodemographic and health-related characteristics

During the first six-month supervised period, falling during the previous 12 months (p = 0.007) was more common in women with high adherence (43 %) than in those with low adherence (30 %). Also, the ability to ride a bicycle at baseline (p < 00.001), mean duration of utility exercises (99.5min vs. 81min, p < 0.05) and that of vigorous exercises (55.1min vs. 48.2min, p < 0.05) were significantly higher among these women (Table 2).

During the second year (unsupervised period), those who purchased a gym card and continued the exercise routine were about one year younger than those who did not purchase it (75.4 vs 76.7; p < 0.001) (Table 3). They also had higher self-reported mean durations of single strength (60.2min vs 49.2min; p < 0.05), endurance (89.0min vs 77.1min; p < 0.01) and utility exercises (108.8min vs 87.4min; p < 0.05) (Table 2). In addition, they had less often no history of strength training (35 % vs. 46 %; p < 0.01) (Table 2).

### 3.3 Adherence related to mental health and functioning

During the first six-month supervised period, fear of falling itself was not related to adherence, but fear of falling that did not limit day-to-day mobility was related to high adherence (86 % vs 77 %; p < 0.05). Also, women with better social well-being such as having someone who loves (94 % vs 86 %; p = 0.01) and loving someone (96 % vs 90 %; p < 0.01) belonged more often to the high adherence group. This was true also with internal hostility (i.e. being easily irritated) (85 % vs 77 %; p < 0.05), whereas external hostility (i.e. losing temper easily) was related to lower adherence (3 % vs. 21 %; p < 0.05). Depression, perceived stress, resilient coping, optimism, or life satisfaction were not significantly related to adherence (Table 4).

During the second year (unsupervised period), women who did not purchase a gym card had more often reported fear of falling than those who purchased it (57 % vs. 44 %; p < 0.05). No other significant differences related to mental health and functioning concerning adherence were observed in this period (Table 4).

### 3.4 Physical tests

During the supervised period, none of the physical performance results were related to adherence (Table 5). During the second year (unsupervised period), higher muscle strength predicted the continuation of the exercise. Those who purchased gym card had higher baseline strength in the isometric left leg extension test (314.5 N vs. 296.02 N; p < 0.05) and in the maximal grip strength test (27.5 kg vs 26.3 kg; p < 0.05) than those who did not continue their exercise. They also performed significantly faster in TUG -test (9.4sec vs 9.9sec; p < 0.05) (Table 5). The associations of single leg stance test, squat test, and body sway analysis with continuing to exercise were not statistically significant.

## 4 Discussion

The majority (∼62 %) of community-dwelling, elderly women (N = 457) randomized in the two-year exercise intervention group had high adherence (>80 % of sessions attended) during their initial six-month free-of-charge, supervised exercise period, whereas only 26 % continued into the second year's low cost, self-sustained period. Active training history was related to better adherence in both periods, but also period-specific predictors were found. In the initial period, except for previous falls, and the ability to ride a bicycle, the predictors of high adherence were related to mental health i.e. having less often fear of falls that limits one's mobility, a loving relationship, and ability to cope with external, but not internal hostility. In the second year, predictors were related to younger age, having less frequent fear of falls, better functional capacity i.e. better strengths (grip and leg extension) and faster TUG -test.

This study addressed the differences in adherence status in women who had been randomized in the exercise intervention RCT group of the KFPS. Thus, the results are not directly comparable to the results of studies addressing predictors of overall participation in physical activities among population-based cohorts. According to van Heuvelen et al. (2015) [28], in RCTs, non-participation has been associated with lower levels of cognitive functioning, more depressive symptoms, and lower levels of daily physical activities. Also, participants in the KFPS exercise intervention differed to some degree from the non-participants and non-invited subjects with respect of mental and physical health, functional capability, place of residence, and sociodemographic status [29].

In exercise interventions, adherence rates vary widely according to different study populations, duration of intervention, and content of the exercise protocols. Program characteristics such as home-based vs. group-based and aerobic vs. strength training programs seem to affect adherence [30]. According to a meta-analysis by Hong et al., 2008 [31], group-based exercises are more likely to result in higher attendance compared to individual training, but different factors such as exercise program characteristics are likely to contribute as well. Further, adherence rate has been typically defined by counting the proportion of participants attending available sessions or those who complete the entire study programs, or as the proportion of complete dropouts [32]. Thus, there is no golden standard for a “good” or “adequate” adherence rate. Although in previous literature acceptable participation rates have been considered to be somewhere over 80 % (25].

Due to the multifactorial nature of “adequate” adherence rates in different studies, straight comparisons are not feasible. Previously, around 50 % of the elderly who attend exercise programs have been estimated to continue during the first six months [33]. In a more recent review, the mean adherence rate in exercise programs for the elderly lasting over six months was 69 % [34]. Thus, our results are in alignment with these findings as about 60 % of women in our intervention group continued the exercise after the first six months.

In a systemic review of prospective studies assessing adherence to exercise programs, living alone has been associated with better adherence [32], but in the present study, marital or housing status was not. Nevertheless, we reported that social well-being such as a loving relationship, and the ability to control external hostility (i.e. not losing temper easily) but not internal hostility (being easily irritated) were all related to high adherence in the initial, free-of-charge, supervised training period. Losing one's temper more easily may hinder participation or lead to dropping out from group exercise sessions, whereas problems with internal hostility may advance high adherence after having started to exercise due to the perceived tempering effect of physical activity. According to a meta-analysis by Ricke et al. (2023) [35] concerning home-based exercise therapy, high adherence was associated with self-efficacy, exercise history, perceived behavioral control, motivation, and physical health. Less depression, comorbidities, and fatigue were also predictors of better adherence.

One-third of people aged over 65 may fall at least once a year [36]. Previous falls could be thought to motivate aging women to high participation and adherence to exercise intervention, especially if they have received health education concerning falling as they had in the KFPS. Indeed, the association between previous falls and high adherence was seen in our study, but only in the initial six-month exercise period. Secondly, the fear of falling was a significant predictor of non-adherence in the second year. This should encourage further integration of fall-related education added to exercise intervention to maintain and motivate a long-term active life among the elderly.

Those with high adherence to the protocol training sessions also had more additional visits outside supervised sessions during the whole no-cost period (months 1–12). Similar results were seen among those who purchased access for the second year compared to those who did not (Table 5). This indicates that the same factors associated with high adherence to exercise intervention, seem to increase willingness to independent exercising in older women. When viewing the amount of independent exercise in this study, the proportion of women who purchased low-cost access for the second year and continued training by themselves remained quite low, which limits the reliability of the analysis to some degree. Purchasing access to training facilities doesn't guarantee regular exercise per se. However, participants who continued exercising after the first year, also had moderately high number of visits during the second year. Therefore, purchasing access remained the most reliable indicator of long-term training. Nevertheless, assumption involving training intensity could not be made on purchase alone. Therefore, no additional analysis on training intensity were conducted while possible variations of physical activity remain unknown. In addition, the participants in this study are elderly. Their health status may change rapidly over a short period, which may appear as poor adherence or dropping out due to health problems. This may limit the accuracy of our results. The review article by White et al. (2005) [37] reported that training programs with content varying by time may be beneficial considering training adherence. The baseline questionnaire in our study did not focus on specific factors such as personal attitudes and previous experiences towards physical training which can contribute to adherence as well. In addition, the current study population sample consisted of females, which limits the generalizability of these findings in males.

Our study aimed to provide further insight into the predictors of adherence in exercise intervention among aging women. As a review by Collado-Mateo et al. (2021) [38] shows, several factors could be assessed before starting the program to achieve better adherence rates. These should include physical and mental characteristics and personality features. However, individuals who could receive the most benefits from exercising, are more likely participate the least [39]. Thus, the biggest challenge in promoting and maintaining health programs at an older age is to get those with poorer physical or mental health to participate, including those with emerging social or cognitive problems. Promoting mental health and well-being among the elderly might increase their willingness to participate in exercise programs. The effect of group exercise and social contacts can also be seen to result in higher adherence. Professionals who work with the elderly exercise activities should consider group exercise programs as one of their priorities to maximize adherence. In addition, supportive strategies for encouraging the elderly with mood or psychosocial challenges should be planned to better engage them in such exercise activities and maintain their functional capacity.

In conclusion, an active training background is associated with adherence to an exercise RCT. Better mental health is associated with higher adherence to supervised exercises, while better functional capacity predicts independent continuation afterwards. Further research on prerequisites for persistent long-term adherence to physical activity among the elderly is needed. Especially among those at risk of sedentary lifestyle and its adverse health outcomes.

### 4.1 Strengths and limitations

This study investigated long-term adherence during the two-year intervention. In most studies that have studied adherence, the duration of interventions has been between 6 and 12 months [34]. In addition, this study extensively investigated factors such as physical, mental, sociodemographic and lifestyle. Adherence data was obtained from both supervised and self-sustained exercising.

The fact that the original study and the baseline questionnaire weren't primarily developed for the investigation of adherence can be considered a limitation of this study. Therefore, some adherence-related factors that might have been relevant to the study results were not available for analysis. Further, the number of women who purchased gym access for the second year remained quite low which limit's reliability of the analysis to some degree.

## Data availability statement

The anonymized data underlying this article will be shared on reasonable request according to ethical, privacy, and legislation issues.

## Declaration of ethics

KFPS studies have been reviewed and approved by the Ethics Committee of the Hospital District of North Savo and Kuopio University Hospital on 31 January 2014, 29 April 2016 and 16 December 2016. (Diary number: 221/13.02.00/2015). Written informed consent was obtained from every patient. All regulations and measures of ethics and confidentiality are handled in accordance with the Declaration of Helsinki.

## CRediT authorship contribution statement

**J. Laakso:** Writing – original draft, Visualization, Formal analysis. **J. Kopra:** Writing – review & editing, Methodology, Investigation, Conceptualization. **H. Koivumaa-Honkanen:** Writing – review & editing, Methodology, Investigation, Funding acquisition, Conceptualization. **J. Sirola:** Writing – review & editing, Methodology, Investigation, Funding acquisition, Conceptualization. **R. Honkanen:** Writing – review & editing, Methodology, Investigation, Funding acquisition, Conceptualization. **H. Kröger:** Writing – review & editing, Methodology, Investigation, Funding acquisition, Conceptualization. **T. Rikkonen:** Writing – original draft, Project administration, Methodology, Investigation, Funding acquisition, Formal analysis, Conceptualization.

## Declaration of competing interest

The authors declare that they have no known competing financial interests or personal relationships that could have appeared to influence the work reported in this paper.
